# Ocean-Bottom Seismographs Based on Broadband MET Sensors: Architecture and Deployment Case Study in the Arctic

**DOI:** 10.3390/s21123979

**Published:** 2021-06-09

**Authors:** Artem A. Krylov, Ivan V. Egorov, Sergey A. Kovachev, Dmitry A. Ilinskiy, Oleg Yu. Ganzha, Georgy K. Timashkevich, Konstantin A. Roginskiy, Mikhail E. Kulikov, Mikhail A. Novikov, Vladimir N. Ivanov, Elena A. Radiuk, Daria D. Rukavishnikova, Alexander V. Neeshpapa, Grigory O. Velichko, Leopold I. Lobkovsky, Igor P. Medvedev, Igor P. Semiletov

**Affiliations:** 1 Shirshov Institute of Oceanology, Russian Academy of Sciences, 36, Nakhimovskiy Prospekt, 117997 Moscow, Russia; kovachev@ocean.ru (S.A.K.); dilinskiy61@mail.ru (D.A.I.); ganzhaoy@mail.ru (O.Y.G.); tim@ocean.ru (G.K.T.); roginskiy@list.ru (K.A.R.); meksonesk@gmail.com (M.E.K.); mihail.novikow0@gmail.com (M.A.N.); ivanov.vl-mir@yandex.ru (V.N.I.); elena.radiuk@yandex.ru (E.A.R.); drukavishnikova@gmail.com (D.D.R.); llobkovsky@ocean.ru (L.I.L.); medvedev@ocean.ru (I.P.M.); 2 Moscow Institute of Physics and Technology, 9, Institutsky lane, 141700 Dolgoprudny, Russia; egorov.ivan83@gmail.com (I.V.E.); AlexN@r-sensors.ru (A.V.N.); 3 V.I. Il’ichev Pacific Oceanological Institute, Far Eastern Branch of the Russian Academy of Sciences, 43, Baltijskaya St., 690041 Vladivostok, Russia; ipsemiletov@alaska.edu; 4 Kuban State University (KubSU), 149, Stavropolskaya St., 350040 Krasnodar, Russia; grishavel@mail.ru; 5 National Tomsk Polytechnic University, 30, Lenina Prospekt, 634050 Tomsk, Russia; 6 National Tomsk State University, 36, Lenina Prospekt, 634050 Tomsk, Russia

**Keywords:** ocean-bottom seismograph, molecular–electronic transfer seismometer, seismic hazard assessment, teleseismic signal, local microearthquake, ambient seismic noise, site response analysis, Laptev Sea, Arctic region

## Abstract

The Arctic seas are now of particular interest due to their prospects in terms of hydrocarbon extraction, development of marine transport routes, etc. Thus, various geohazards, including those related to seismicity, require detailed studies, especially by instrumental methods. This paper is devoted to the ocean-bottom seismographs (OBS) based on broadband molecular–electronic transfer (MET) sensors and a deployment case study in the Laptev Sea. The purpose of the study is to introduce the architecture of several modifications of OBS and to demonstrate their applicability in solving different tasks in the framework of seismic hazard assessment for the Arctic seas. To do this, we used the first results of several pilot deployments of the OBS developed by Shirshov Institute of Oceanology of the Russian Academy of Sciences (IO RAS) and IP Ilyinskiy A.D. in the Laptev Sea that took place in 2018–2020. We highlighted various seismological applications of OBS based on broadband MET sensors CME-4311 (60 s) and CME-4111 (120 s), including the analysis of ambient seismic noise, registering the signals of large remote earthquakes and weak local microearthquakes, and the instrumental approach of the site response assessment. The main characteristics of the broadband MET sensors and OBS architectures turned out to be suitable for obtaining high-quality OBS records under the Arctic conditions to solve seismological problems. In addition, the obtained case study results showed the prospects in a broader context, such as the possible influence of the seismotectonic factor on the bottom-up thawing of subsea permafrost and massive methane release, probably from decaying hydrates and deep geological sources. The described OBS will be actively used in further Arctic expeditions.

## 1. Introduction

Seismic hazard assessment and seismic zonation are extremely important and are among the most complicated problems of seismology. These problems are relevant because of the intensification of construction in seismically active areas, including difficult to access and sparsely populated areas. This also applies to the Russian Arctic, which is now being actively developed: oil-and-gas terminals, extracting platforms, and military bases are being built. The vast Arctic shelf zones are not depicted on the normative maps of the general seismic zoning of Russia [1]. The lack of knowledge also applies to geohazards related to seismicity, such as soil liquefaction, underwater landslides, tsunamis, massive methane seepage from the sea bottom, etc. At present, the climate warms twice as fast in the Arctic region and top sea level stands, for about 5–6 thousands years longer than during the previous warm geological epochs [2]. This causes the progressive subsea permafrost thawing and consequent massive methane release in the Russian Arctic seas, especially in the East Siberian Arctic Shelf (the broadest and shallowest shelf in the World Ocean) which represents >80% of the subsea permafrost and mega-pool of hydrates [3,4,5]. Numerous geohazards could be related with progressive methane release, including impact on infrastructure of the Northern Sea Route, gas and oil under-water pipes, and general gas and oil exploration.

Seismic hazard assessment is a set of theoretical and instrumental methods aimed at solving a wide range of tasks, from describing regional-scale tectonic processes in the study area to assessing the influence of local conditions on the propagation of seismic waves at the construction sites [6]. Instrumental studies using local networks of seismic stations are the most important source of information to solve both fundamental and applied seismological problems. The main tasks of local instrumental observations include registration of remote and local earthquakes, seismic noise analysis, obtaining the location and activity of seismogenic structures, obtaining deep structure, description of seismic regime, assessment of local site amplification, and others [7].

The peculiarities of work at sea, especially in the Arctic, lead to the need for special approaches to the design of the ocean-bottom seismographs (OBS) and to their deployment on the seabed. Since it is impossible to be sure that the seabed will be substantially flat at the deployment site of the OBS, the allowable inclination for seismic sensors should be wide enough. Sensors are required to be tolerant to unfavorable conditions of transportation, deployment, and operation. It is also difficult to accurately determine the orientation of the sensors on the seabed relative to the pole, even with digital compass modules. Therefore, it is necessary to install a network of OBS or use them in combination with on-land stations.

Due to the fact that GPS synchronization of the internal clock is possible only before the deployment of OBS on the seabed, and re-synchronization when the stations dismantle is not always possible, depending on the experiment duration, then their accuracy should be high due to the use of chip-scale thermo-stated quartz or atomic clocks. Many seismological tasks require long-term deployments, resulting in the need for a corresponding power source. The first Arctic experiments showed that the ice cover significantly reduces the seismic noise caused by the wind waves [8]. Thus, the OBS must be functional for at least the ice-covered time period. At the same time, it is obvious that work in the Arctic imposes requirements on the stations for the stable operation of all equipment at low temperatures.

The presence of ice cover at the Arctic seas for most of the year leads to the need for considering the probability of damaging the OBS by icebergs and stamukhi in shallow shelf waters. The Arctic shelf seabed is dotted with ice plough marks [9,10]. Therefore, the year-long installation of OBS at sites with a sea depth less than 40 m is largely unsafe.

Long-term installations increase the risk of equipment loss. This leads to the need to develop reliable mechanisms for OBS recovery: hydroacoustic connection, release devices, and possible trawling schemes. The weight of the equipment also becomes an important parameter, as well as its cost. In addition to the above, the solution of seismological problems imposes significant requirements on the width of the frequency range, sensitivity, and dynamic range of the sensors and recording equipment.

The purposes of the present paper are to introduce the architecture of several modifications of the OBS based on broadband molecular–electronic transfer (MET) sensors and to demonstrate their applicability in solving different seismic hazard assessment tasks. To do this, we used the first results of several pilot deployments of the OBS developed by the Shirshov Institute of Oceanology of the Russian Academy of Sciences (IO RAS) and IP Ilyinskiy A.D. in the Laptev Sea, that took place in 2018–2020.

The MET sensor has a number of significant advantages, such as increased reliability in operation, absence of special transportation conditions, low energy consumption, wide temperature range, insensitiveness to installation angles, and no need to fix or center the mass [11]. Recently, the use of this type of sensors has become more frequent for various monitoring tasks, including in the Arctic seas [12,13,14,15,16]. As it will be shown below, the use of the sensors of this type in OBS also demonstrated their effectiveness in solving seismic problems, such as registration of remote and local earthquakes, seismic noise analysis, and assessment of local site amplification, under the severe conditions of the Arctic seas.

## 2. Instrumentation

### 2.1. Broadband MET Seismometers

**Figure 1:**
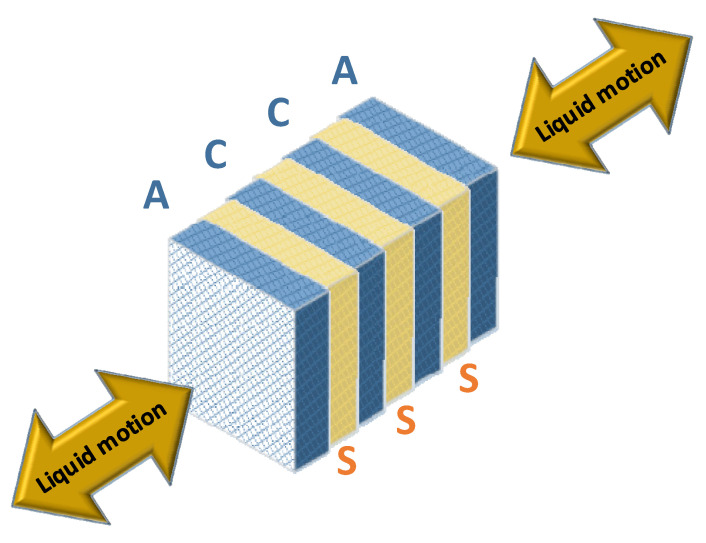
The sensing element in electrolyte (denoted: A—anodes, C—cathodes, S—spacers).

The OBS are equipped with two types of broadband MET sensors depending on the modification: CME-4111 (120 s) and CME-4311 (60 s), developed by R-sensors, Dolgoprudny, Russia [17]. An electrochemical sensing element of CME-4111/4311 seismometers consists of two pairs of platinum mesh electrodes that form a four-electrode anode-cathode-cathode-anode (ACCA) system. To protect the adjacent electrodes from contact, permeable dielectric spacers are used. The whole assembly is immersed in an iodine–iodide electrolyte that can move freely in one direction along a round or square channel through the electrode-spacer system (see Figure 1).

**Figure 2:**
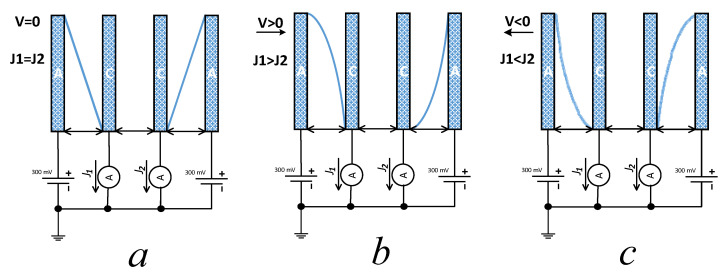
The active component concentration distribution in the electrolyte ((**a**) steady state, (**b**) and (**c**) moving to the right and left, respectively).

As soon as a small potential difference, say 0.2–0.6 V, is applied to both pairs of electrodes, a reversible chemical reaction of oxidation of iodine at the anodes:

(1) $$I_{3}^{-}+2e^{-}\to \;3I^{-}$$

and a reduction of iodine at the cathodes:

(2) $$3I^{-}-2e^{-}\to {\;I}_{3}^{-}$$

take place [18]. This results in a charge transfer between anodes and cathodes via the electrolyte ions in the solution. The above-mentioned potential is sufficient to bring the electrochemical process to saturation, so the current becomes limited by the volumetric rate of supply of the active component to the electrodes rather than the applied potential. In turn, the supply rate is determined by the ion diffusion in the stationary electrolyte. In the steady state, a symmetric concentration distribution of the active component is observed, which results in an equality of the currents *J_1_* and *J_2_* flowing through the pairs of electrodes (Figure 2a).

As the electrolyte starts moving through the sensing element, the convective flow is always directed from a cathode to an anode for one pair of electrodes and in the opposite direction for another. Therefore, the concentration distribution of the active component shows asymmetry, which results in a current difference related to the amplitude and the direction of the electrolyte motion (Figure 2b,c) [19].

Being employed in the condition of saturation, molecular–electronic sensing elements show a high conversion factor that can register the smallest motion exposures, such as those of the natural seismic Earth signals over the noise of accompanying electronic circuitry.

However, the output response of a molecular–electronic sensing element shows neither a velocity-flat nor an acceleration-flat response. The frequency-dependent response gives rise to the task of frequency equalization of the sensing element response. The actual parameters of a sensor depend on its inner geometry and display a noticeable range, even for two identically made sensors of one batch. Therefore, the task of achieving the desired accuracy can only be performed by means of measuring the cell transfer function directly.

**Figure 3:**
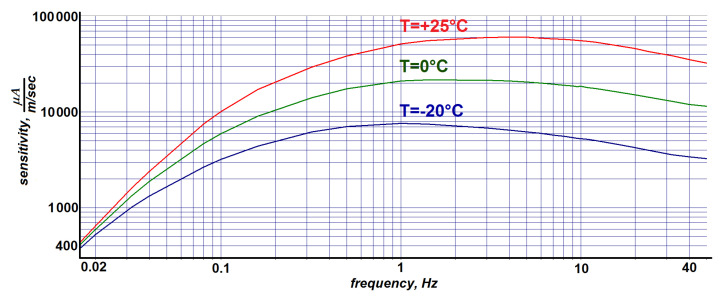
The amplitude-frequency response of a typical sensing element at different temperatures.

Apart from this, the electrochemical reactions show exponential dependence of their rate vs. temperature [20,21]. Due to this dependence and a change of the sensor mechanical parameters over temperature, the sensitivity of sensing elements show frequency-dependent behavior over temperature. Typical velocity vs. frequency responses of a horizontal sensor at three different temperatures are provided in Figure 3. As a result, the task of equalizing the sensor frequency response should contain both frequency-dependent and temperature-dependent elements.

The molecular–electronic sensors’ construction differs whether a sensor is equipped with a force feedback mechanism or not. As a rule, the sensors without a force feedback mechanism are of horizontal type, which is in place in both CME-4111 and CME-4311, with a rigid case made of ceramics. Despite the technical possibility of such improvement, the vertical sensors of CME-4111 seismometers are not equipped with a force feedback as well. This was done intentionally to achieve the lowest power consumption. The compensation of temperature and frequency dependence for no-feedback sensors can only be performed by means of a combination of low-pass and high-pass filters with temperature-dependent elements.

The force feedback mechanism in the vertical sensor of CME-4311 contains a firmly fixed permanent magnet and a coil, which in turn is fixed on a moving part of a sensor. The force feedback mechanism is provided by an interaction of the magnet with a current flowing in the coil. So, the circuitry of a sensor with a force feedback contains a current amplifier, which on the one hand allows to improve the stability and the evenness of the frequency response, but on the other hand, requires more power consumption to keep the current in the feedback coil. The introduction of a force-feedback mechanism into a sensor allows reducing the requirements that are imposed on the precision of the frequency and the temperature stabilization.

Since the power consumption is the most important when designing autonomous subsea systems, there is a trade-off between the precision of an instrument and its power demands. A CME-4111, which is a three-component, no-feedback broadband seismometer, consumes only 7 mA from a 12-V power source, while the same size CME-4311, which has just one force-feedback and two no-feedback components, needs 8.5 mA in a steady state. However, the vertical component of CME-4311 has a ±0.5 dB tolerance in the middle of a passband and ±1 dB at the slopes, compared to ±1 and ±2 dB of CME-4111, respectively. The comparison shows that the improvement of precision has been achieved by introduction of a force feedback.

**Figure 4:**
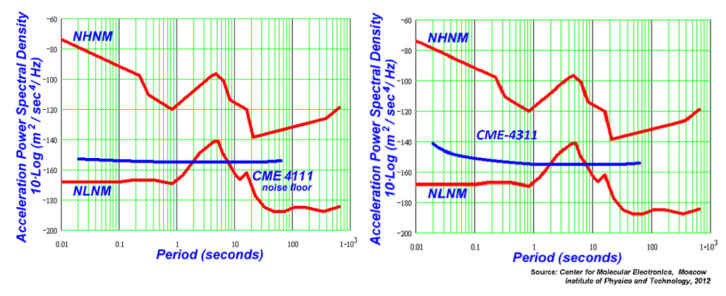
The self-noise of CME-4111 (**left**) and CME-4311 (**right**) seismometers.

The bandwidths of the sensors employed in the study are 0.0083 (120 s) to 50 Hz for CME-4111 and 0.0167 (60 s) to 50 Hz for CME-4311. The noise performance of both seismometers is almost identical, with a slight rise of noise density of the vertical channel of CME-4311 in higher frequencies (Figure 4). This rise of noise density is of electronic amplifier origin and results from a higher gain in the forward amplification circuit needed for a system with a negative feedback. The parameters of both seismometers are listed in Table 1.

**Figure 5:**
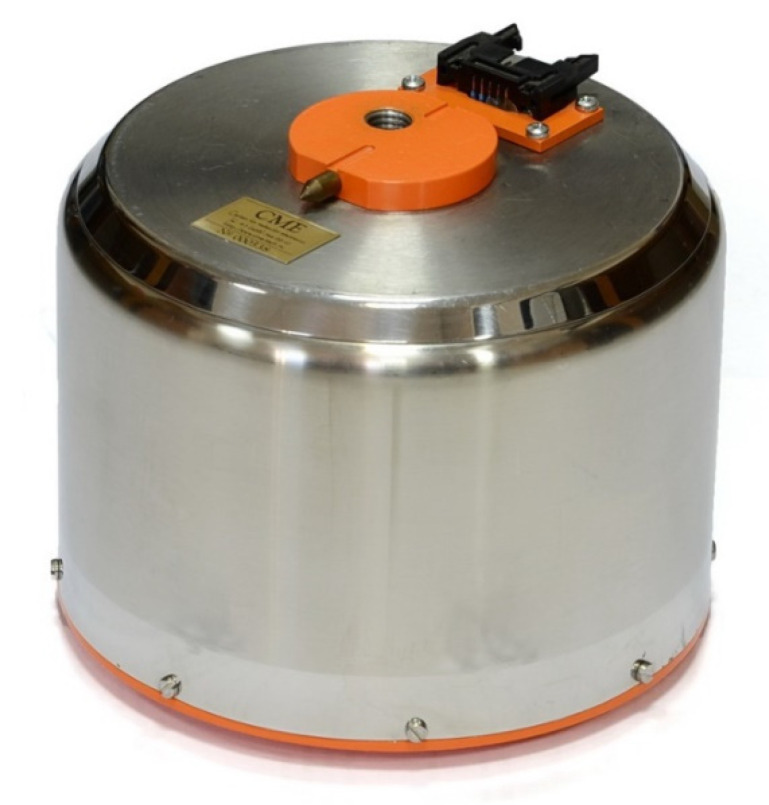
The CME-4111/CME-4311 seismometer in an optional stainless-steel case (Courtesy of R-sensors LLC).

Both seismometers optionally have the same type and size, light stainless-steel case, and are pin-to-pin compatible (Figure 5).

### 2.2. OBS Modifications for Shallow Waters

**Figure 6:**
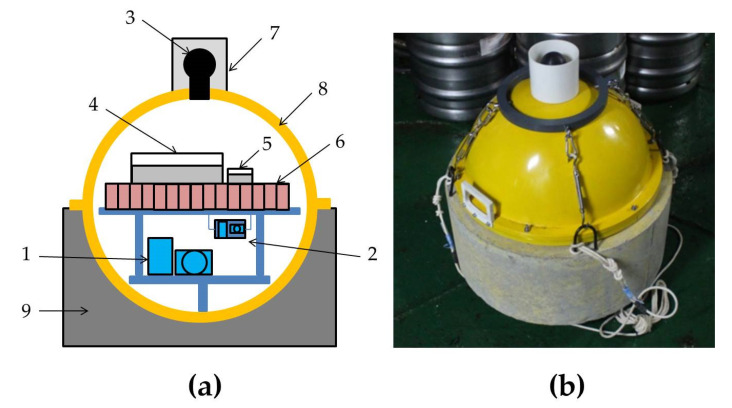
The design of the MPSSR ocean-bottom seismograph developed by the IO RAS (**a**) and its external view on the R/V Akademik Mstislav Keldysh, autumn 2018 (**b**). (1) Three-component broadband seismometer CME-4311, (2) three-component short-period seismometer (SV-10 and SH-10) placed in gimbal, (3) Hydrophone 5007m, (4) recorder URS-S, (5) digital compass module, (6) batteries block, (7) protective half-cover for hydrophone, (8) duralumin sphere, (9) concrete ballast.

The MPSSR ocean-bottom seismograph developed by the IO RAS is suitable for a wide range of tasks, including seismological monitoring, active and passive seismics and high-resolution seismoacoustic investigations. The design of the MPSSR and its external view are presented in Figure 6. The MPSSR is equipped with two three-component seismometers and a hydrophone. The first three-component seismometer is broadband and consists of CME-4311 MET sensors produced by R-sensors, with a frequency range of 0.0167–50 Hz. The second three-component seismometer is short-period and consists of SV-10 and SH-10 classic electromechanical geophones, with a frequency range of 10–200 Hz (analogy of GS-20DX), placed in gimbal. The Hydrophone 5007 m included in the package was also developed by the IO RAS, Moscow, Russia and has a frequency range of 0.04–2500 Hz. The basic parameters of the Hydrophone 5007 m are presented in Table 2.

The recorder URS-S is based on the ARM microcontroller STM32F103. It is supposed to be a part of autonomous devices intended for seismic research. It can also be used wherever multi-channel acquisition of analog signals with a reference to absolute time is required, with low power consumption. Table 3 shows the main characteristics of the recorder URS-S. The features of the recorder include its relatively low power consumption and the presence of a built-in high-precision thermo-stated reference frequency generator MXO37/8P [22], in combination with a GPS interface, which makes it possible to time-tie the received records to absolute time. The Trimble AcutimeTM GG antenna is used for receiving a synchronizing GPS signal. The sample rate can be chosen from 20 up to 800 Hz. The use of a combined anti-aliasing filter significantly increases the dynamic range of the recorder in relation to the dynamic range of the ADC. An ordinary SD memory card with the capacity of up to 64 Gb is used for data storing. The recorder is powered from a single positive-polarity power supply with the conversion into voltages required for the recorder circuits to operate. The power supply voltage is 6.5–32 V, and the consumption in the recording mode is about 20 mA, at a supply voltage of 12 V. In addition, the recorder generates the voltages required for the operation of external analog sensors, which are the sources of the recorded signals, as well as the supply voltage of the GPS receiver. A round and flat battery package is located in the central part of the station and can be equipped with various types of alkaline or lithium batteries.

The MPSSR is also equipped with a separate digital compass module based on the digital three-axis magnetometer, MAG3110. The module has its own internal built-in memory and a separate power supply, with the voltage of 9 V and the battery capacity of 200 mAh. It only works for the first 12 h after turning on the OBS to capture the moment when the OBS touches the seabed and to save the orientation data.

**Figure 7:**
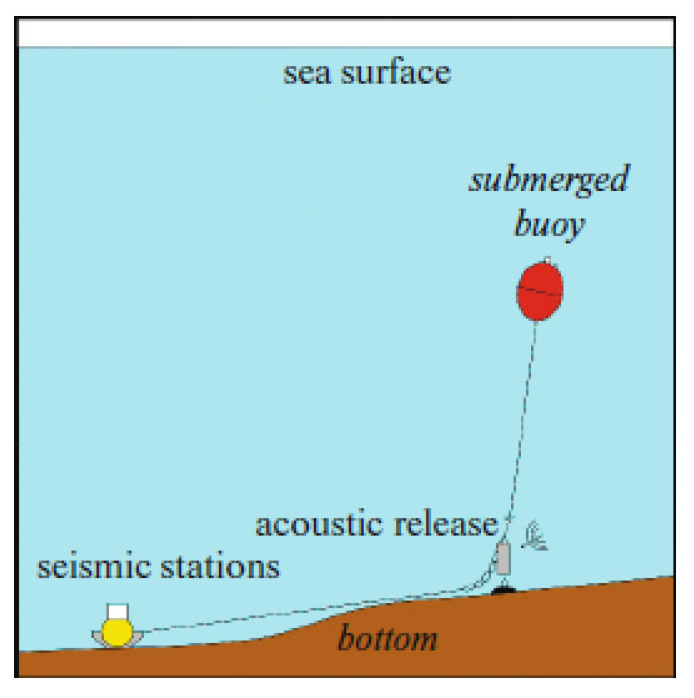
MPSSR ocean-bottom seismograph deployment scheme on the shallow shelf with a submerged buoy.

The MPSSR has duralumin spherical housing with a diameter of 444 mm designed for depths up to 3000 m. The housing is rigidly attached to the concrete ballast to improve the traction on the seabed. The current equipment is not self-pop-up and the deployment in shallow waters is conducted with the use of external acoustic release and buoys, according to the deployment scheme presented in Figure 7. The deployment scheme allows trawling a rope laid on the seabed between the OBS and the ballast-buoy system if the acoustic release does not work after long-term operation. Thus, the current equipment and the deployment scheme imply work on the shelf at depths of no more than 100 m.

There is also a short-period Typhoon ocean-bottom seismograph with a frequency band of 1–50 Hz. It is a smaller modification of the MPSSR with the housing diameter of 350 mm and it is equipped with one three-component seismometer CME-3311 (see Table 4) and the Hydrophone 5007 m. The recorder URS-S is also used in it.

When installing on the seafloor, the MPSSR and Typhoon stations are usually accompanied by autonomous wave recorders, which are attached to a rope not far from the OBS. The wave recorder ARW-K14-1 is equipped with a quartz baro-sensitive element, in which the membrane is bent by the action of the column pressure of the liquid, deforming a strong sensitive piezo-element attached to it. Thus, the absolute pressure at the point of measurement can be calculated. The wave recorder is also equipped with a temperature compensation system, which allows the temperature to be measured at the same time as the pressure. The pressure is recorded at a sampling rate of 1 Hz. Another device is a RBR virtuoso3D wave logger [23], equipped with a Keller pressure sensor, which is also based on the use of a piezoelectric quartz sensor as a baro-sensitive element. It records the pressure values of the water column and translates them into the variable depth of the location. The recording frequency is 1 Hz.

### 2.3. OBS Modification for Deep Waters

**Figure 8:**
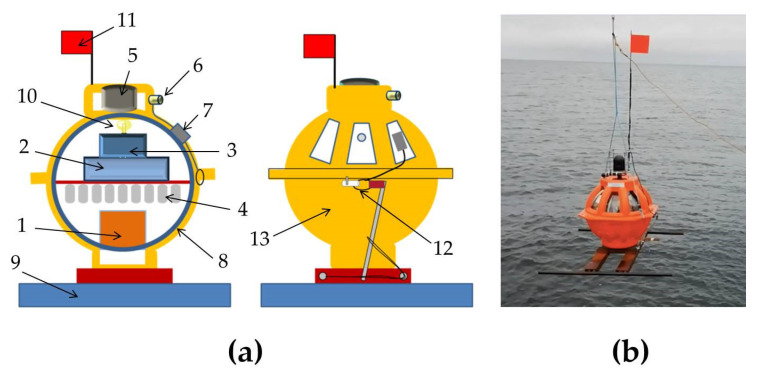
Design of the GNS-C ocean-bottom seismograph developed by IO RAS and IP Ilinskiy A.D. (**a**) and its external view on the R/V Akademic Mstislav Keldysh, autumn 2018 (**b**). (1) Three-component broadband seismometer CME-4111, (2) recorder GNS, (3) acoustic modem, (4) batteries block, (5) acoustic hydrophone, (6) seismic hydrophone, (7) penetrator, (8) glass spherical housing with diameter 430 mm, (9) anchor, (10) lamp, (11) flag, (12) anchor release, (13) plastic case.

Unlike the MPSSR and Typhoon models described above, the GNS-C model is used for scientific applications of the IO RAS in deep waters. It was developed by the IO RAS and IP Ilinskiy A.D. The GNS-C employs the 120 s MET sensor CME-4111, produced by R-sensors. GNS-C is a self-pop-up OBS for the water depths ranged up to 6000 m and is suitable for studying seismicity, tectonics and deep structure, down to the middle mantle. The design of the GNS-C and its external view are presented in Figure 8. The general characteristics of the GNS-C are shown in Table 5. The notable capabilities of the station are:

- Multiple seabed deployment/recoveries without opening or recharging the node.
- Automatic clock synchronization with GPS signals through the instrument case. Built-in GPS receiver, activated automatically after surfacing node from the seabed.
- Wireless on/off power switch. No need to open the node case after the transport and seabed recovery, all preparation and tests before deployment could be performed on a vessel deck.
- Wireless user interface. Fast data download after the node retrieval to a ship deck.
- Automatic and manual tests of power supply, seismic recorder and acoustic release.

In addition to the three-component seismometer CME-4111, the station is equipped with a low-frequency hydrophone produced by the Experimental Design Bureau of Oceanological Engineering, Russian Academy of Sciences (EDBOE RAS). A brief technical specification of the hydrophone is presented in Table 6.

The OBS has a GNS-4-channel seismic recorder with a 24-bit ADC converter for each channel. Table 7 shows the main technical characteristics of the GNS recorder. There is a built-in, high-precision thermo-stated reference frequency generator MXO37/8 and GPS interface with the use of a Trimble Silvana or uBlox UC530M receiver.

Hydroacoustic connection is maintained with the onboard autonomic acoustic unit, powered from 220 V or from internal batteries in a waterproof case. The acoustic link range is up to 10 km, and the frequency range of an acoustic link is 9–13 kHz.

GNS-C is also equipped with additional sensors, such as two temperature sensors, a current measurement sensor, a 3D-compass module, a digital pressure sensor and an analog pressure manometer. Additional OBS sensors are shown in Table 8.

## 3. OBS Deployment Case Study in the Arctic and the First Results

### 3.1. Scientific Cruises to the Laptev Sea

The Laptev Sea is one of the most interesting regions for complex scientific research. It is the most seismically active water area among the Russian Arctic seas [24]. In addition, in the Laptev Sea and East-Siberian Sea, a large number of methane outflows from the seabed have been found, both on the shelf and on the continental slope [3,4,5]. Methane, in the case when it has an endogenous, deep origin, can come from great depths along faults and reach the level of occurrence of gas hydrates, where it mixes with near-surface methane. At the same time, weak microearthquakes occurring in such areas indicate active faults. Moreover, seismotectonic events can affect the intensity of gas vents [25,26,27,28,29,30]. The study of such phenomena is part of the geohazard assessment, including the purely seismic hazard assessment.

Since 2018, the program of seismological research has been included in a series of scientific cruises to the Laptev Sea on the R/V Akademik Mstislav Keldysh organized by the V.I. Ilichov Pacific Oceanological Institute of the Far Eastern Branch of the Russian Academy of Sciences and the IO RAS. The seismological work program has been aimed to determine the seismic and seismotectonic characteristics of the Laptev Sea region in the context of the relationship of the tectonic processes with the discharge of bubble methane from the bottom by registering local microearthquakes, remote teleseismic events and ambient seismic noise on the shelf and the continental slope of the Laptev Sea. In addition, new data on seismicity and present tectonics of the Laptev Sea region are extremely necessary for detailed seismic hazard assessment of the region.

**Figure 9:**
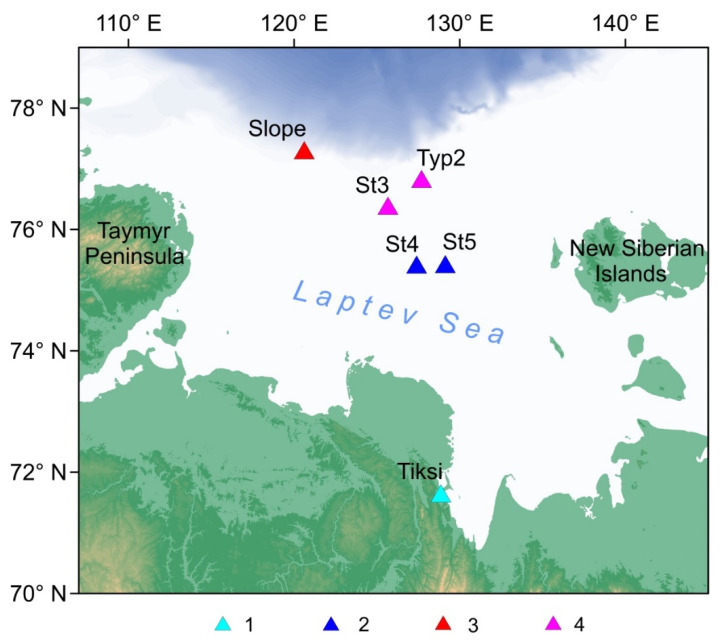
The OBS long-term deployment sites in the Laptev sea. (**1**) Permanent TIXI broadband seismic station (included in Global Seismograph Network [31]), (**2**) the OBS on the inner shelf deployed in the AMK-73 cruise (2018), (**3**) the OBS on the slope deployed in the AMK-73 cruise (2018), (**4**) the OBS on the outer shelf deployed in the AMK-78 cruise (2019).

During the expeditions, the OBS were deployed for a year on the shelf and the continental slope of the Laptev Sea (the technical goal of the seismological work program). By now, the records of two one-year campaigns have been obtained. Figure 9 shows the long-term deployment sites for the AMK-73 cruises (2018) and the AMK-78 cruise (2019). The locations, types and operation periods of the OBS are shown in Table 9.

### 3.2. Recording of Ambient Seismic Noise in the Laptev Sea

In the study of seismic noise on the records of the OBS, it has been found that the ambient noise on the seabed significantly depends on the wind waves and the ice-cover conditions both on the shelf and the continental slope. We compared spectrograms of the signals obtained by different sensors of the OBS, signals obtained by the wave recorders and ice concentration curves derived from the reanalysis data.

Continuously, every second sea level records have been formed based on the data from the ARW-K14-1 and the RBR virtuoso3D tide gauges for 365 and 363 days, respectively. Then, we obtained the data on the intra-annual variability of the level fluctuations in the central part of the Laptev Sea shelf in 2018–2019 (ARW-K14-1) and in the northern part of the Laptev Sea shelf in 2019–2020 (RBR virtuoso3D). To analyze the dependence of the short-period level fluctuations (wind waves, swell) on ice cover, spectrograms of annual sea level records in the range of periods from 2 to 20 s have been constructed. The spectrograms of annual sea level records have been calculated with Welch’s method [32], using the Kaiser–Bessel spectral window in two days, with 50% overlap. Daily ice concentration data have been provided by the European Organization for the Exploitation of Meteorological Satellites (EUMETSAT), with a grid resolution of 25 km [33].

**Figure 10:**
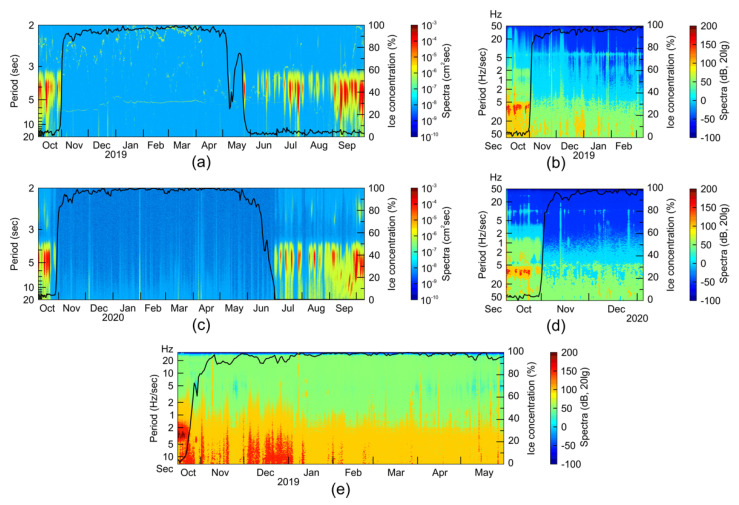
The spectrograms of the ambient seismic noise obtained from the records of: (**a**) wave recorder ARW-K14-1 (2018–2019) for the vicinity of the St4 and St5 sites (see Figure 9), (**b**) horizontal MET sensor CME-4311 (2018–2019) for the site St5, (**c**) wave recorder RBR virtuoso3D (2019–2020) for the vicinity of the St3 and Typ2 sites, (**d**) vertical MET sensor CME-4311 (2019–2020) for the site St3, (**e**) the hydrophone EDBOE RAS for the site Slope (2018–2019). Black solid line—ice concentration (%) at the deployment sites according to [33].

Figure 10 shows the spectrograms of the wave recorders’ data and ambient seismic noise obtained from some channels of the OBS operating in two campaigns, 2018–2019 and 2019–2020. It can be seen from the tide gauges’ records that ice-cover smooths out the wind waves (spectral periods 3–8 s) above the deployment sites. Spectrograms of the horizontal and vertical CME-4311 sensors show that the wind waves in ice-free time periods cause pronounced seismic noise, with two clear spectral period bands: 5–10 s (primary microseisms caused by direct transmission of the surface pressure variations to the seafloor) and 2–3 s (secondary double-frequency microseisms caused by standing wave field) [34]. Ice-cover smooths out the wind waves and therefore significantly reduces the microseisms level. However, more long-period oscillations (spectral periods larger than 30 s) are present in both ice-free and ice-covered time periods and can be caused by infragravity waves [35].

### 3.3. Registration of the Teleseismic Signals

Registration of large remote seismic events is one of the most important purposes of the instrumental seismological studies. Teleseismic signals, both body waves and surface waves, are used for a broad range of tasks, including structural studies by different methods and seismic micro-zonation.

**Figure 11:**
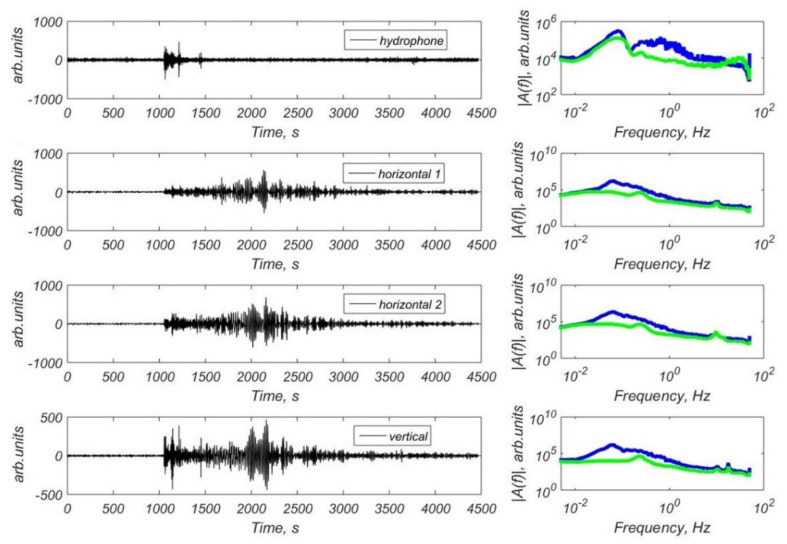
Waveforms and FFT spectra of the earthquake with M = 6.3 that occurred in Alaska (2019-11-24 00:54:01 UTC) [36] obtained by the hydrophone and three-component CME-4311 channels of the MPSSR station at the site St3: blue line—the spectra of the earthquake, green line—the spectra of the seismic noise preceding P-wave arrival.

**Figure 12:**
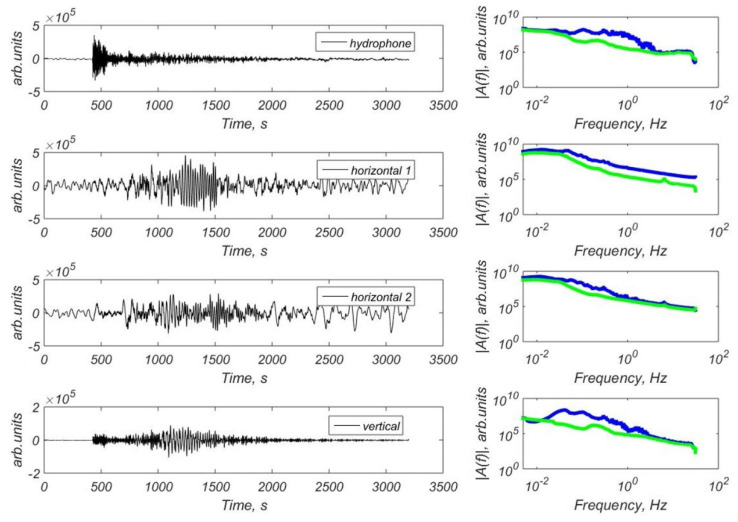
Waveforms and FFT spectra of the earthquake with M = 7.1 that occurred in Alaska (2018-11-30 17:29:29 UTC) [36] obtained by the hydrophone and three-component CME-4111 channels of the GNS-C station at the site Slope: blue line—the spectra of the earthquake, green line—the spectra of the seismic noise preceding P-wave arrival.

We have obtained a significant number of teleseismic signals in the Laptev Sea recorded by the broadband CME-4111 and CME4311 seismic sensors and also by the short-period CME-3311 sensors. Figure 11 shows the example of waveforms and FFT spectra of the earthquake with M = 6.3 that occurred in Alaska (2019-11-24 00:54:01 UTC) [36] recorded by the hydrophone and three-component CME-4311 channels of the MPSSR station at the site St3. Figure 12 shows the example of waveforms and FFT spectra of the earthquake with M = 7.1 that occurred in Alaska (2018-11-30 17:29:29 UTC) [36] recorded by the hydrophone and three-component CME-4111 channels of the GNS-C station at the site Slope. There are clear onsets of body waves and surface waves on the waveforms. Spectra shows a broad frequency band of recorded earthquake signals and seismic noise.

Table 10 contains the list of remote earthquakes that are recorded at St4 and St5 sites, and their P- and S-wave onsets are clear enough to check it by the AK135 Travel Time Tables (campaign 2018–2019). The main information, such as origin time, magnitude, coordinates and depths, has been obtained from the USGS catalog [36]. Table 11 contains the same data for the remote earthquakes that were recorded at St3 and Typ2 sites (campaign 2019–2020).

### 3.4. Registration of the Signals from Local Earthquakes

Local earthquakes’ distribution is an important source of information about present tectonic features of the region, location of seismogentic structures, active faults and their characteristics. Since the local instrumental studies are usually short-term, mainly weak microearthquakes with a magnitude M < 3 and relatively high frequency are recorded. Thus, seismic sensors must have appropriate sensitivity, dynamic range and frequency bands for high-quality recording of both long-period teleseismic signals and short-period signals from local microearthquakes.

**Figure 13:**
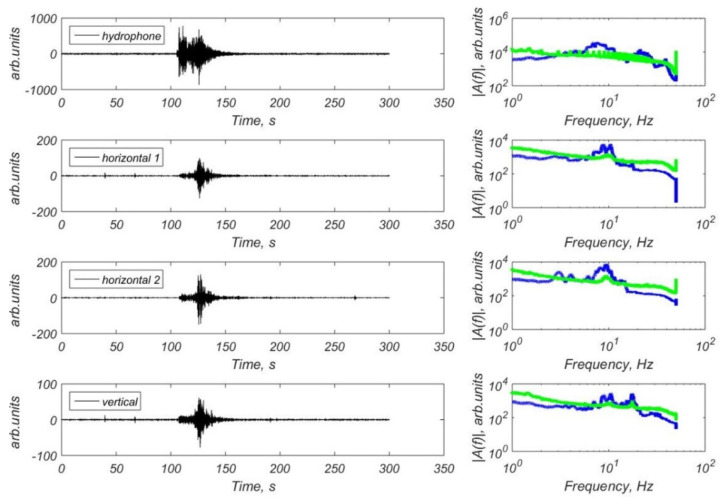
Waveforms (bandpass filter 2–45 Hz applied) and the FFT spectra of the local microearthquake with P-arrival at 2019-11-11 01:58:31 UTC, obtained by the hydrophone and three-component CME-4311 channels of the MPSSR station at the site St3: blue line—the spectra of the earthquake, green line—the spectra of the seismic noise preceding P-wave arrival.

Figure 13 shows the example of waveforms and the FFT spectra of the local microearthquake with P-wave arrival at 2019-11-11 01:58:31 UTC, recorded by the hydrophone and three-component CME-4311 channels of the MPSSR station at the site St3. Since the earthquake signal is buried in long-period seismic noise, the bandpass filter of 2–45 Hz was applied for demonstrating the waveforms.

**Figure 14:**
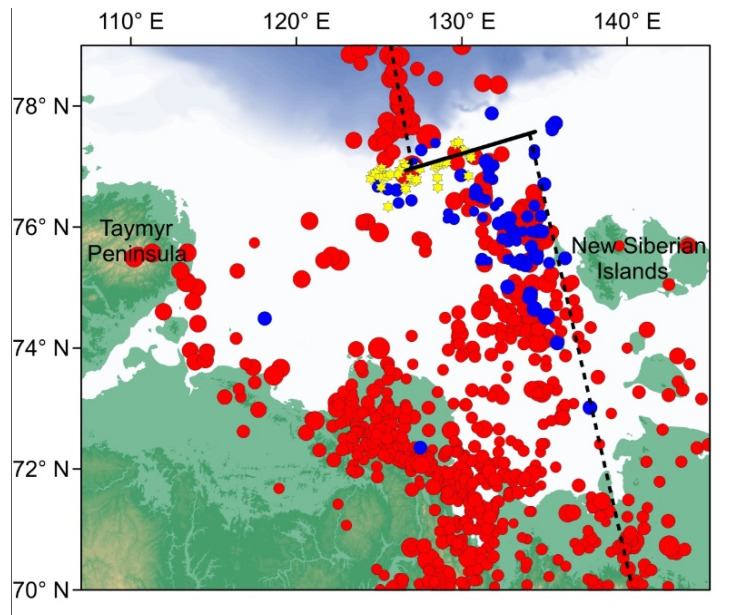
The distribution of the earthquake epicenters in the Laptev Sea region: red circles—the joint regional catalog by ISC [37], USGS [36] and “Earthquakes of Russia” database [38], blue circles—the catalog of the events that were most clearly recorded by the OBS (2018–2019), yellow stars—methane seeps location on the outer shelf [3], dotted line—supposed boundary between the Eurasian and North American tectonic plates [39], solid line—supposed transcurrent fault.

Figure 14 shows the distribution of the earthquake epicenters in the Laptev Sea region obtained from the joint regional catalog (ISC [37], USGS [36], “Earthquakes of Russia” database [38]) and the catalog of the events that were most clearly recorded by the OBS (2018–2019). The OBS data confirm a general shift of the epicenter’s cloud towards the eastern part of the Laptev Sea, which was observed according to regional catalogs. This shift also implies the existence of a transcurrent fault zone. The approximate location of the supposed transcurrent fault on the outer shelf is characterized by a significant number of methane seeps [3]. Thus, this issue is complex and demands as much data as possible and careful seismotectonic interpretation.

**Figure 15:**
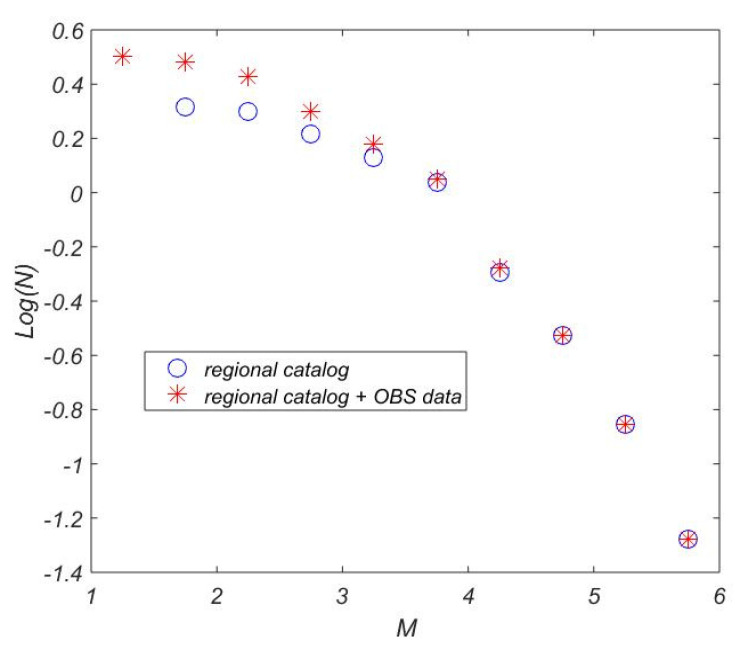
Cumulative recurrence curves for eastern part of the Laptev Sea: blue circles—according to the joint regional catalog by ISC [37], USGS [36] and “Earthquakes of Russia” database [38], red stars—the joint regional catalog combined with the catalog of the events that were most clearly recorded by the OBS (2018–2019).

Figure 15 shows the cumulative recurrence curves for the eastern part of the Laptev Sea according to the joint regional catalog (ISC, USGS, “Earthquakes of Russia” database) and the joint regional catalog combined with the catalog of the events that were most clearly recorded by the OBS (2018–2019). It is clear that the OBS data significantly contribute to completeness of the events catalog in the magnitude range of 1–3.

### 3.5. Site Response Analysis

Site response analysis is a part of seismic microzonation. It is conducted both by empirical methods, numerical modeling and instrumental studies [40]. The site response analysis for the water areas has its peculiarities. Some investigations based on the seafloor seismic records’ analysis show that the vertical components of the seafloor motions are significantly lower than those of the onshore motions near the P-wave resonant frequencies, caused by the water layer above the station [41,42,43].

**Figure 16:**
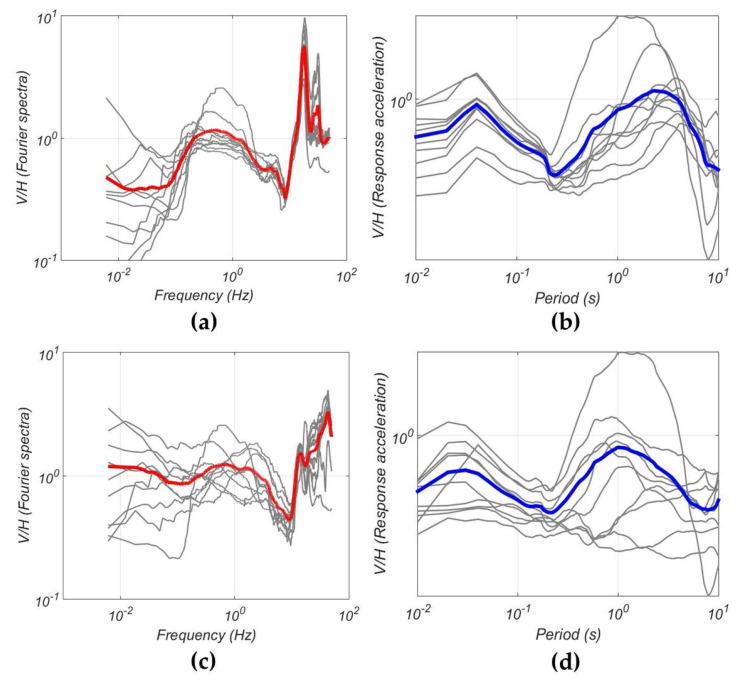
V/H spectral ratio curves of 10 local earthquakes obtained at the St3 site (**a**,**b**) and for the Typ2 site (**c**,**d**). Gray lines—the earthquake spectra, red line—the average curve for the FFT spectral ratios, blue line—the average curve for the response spectral ratios.

The OBS records of local earthquakes in the Laptev Sea allow us to demonstrate this feature of marine site response analysis. Figure 16 shows V/H curves (Fourier spectra and response spectra) for 10 local earthquakes obtained at St3 and Typ2 sites. A similar clear drop of V/H curves at frequencies of 3–10 Hz is observed for both sites, which are located at a significant distance but under a similar water column above the sites (51 and 61 m, respectively). The theoretical estimate of the P-wave resonant frequencies is 5–7 Hz for these depths (obtained by the formula f = c/4H, where c is the sound speed in water and H is the depth of the sea [42]). Thus, the estimated and the observed values are in good agreement.

## 4. Discussion and Conclusions

In this article, we have highlighted various seismological applications of the OBS based on the broadband MET sensors CME-4311 (60 s) and CME-4111 (120 s). There are several modifications of the OBS developed by the IO RAS and IP Ilinskiy A.D., depending on the permissible depths and frequency ranges. The OBS records obtained during the pilot installations in the Laptev Sea were used to demonstrate the applicability of the OBS for seismic hazard assessment tasks in the Arctic seas.

Two field campaigns in 2018–2019 and in 2019–2020 resulted in high-quality seismic records. When using the standard alkaline battery packs, we have obtained 3–5 months of recordings. Using lithium batteries allows for making 7–8 months of recordings. It turned out that the OBS recording capabilities on the Arctic shelf and the upper slope are highly dependent on the level of ambient seismic noise, which, in turn, is influenced by the wind waves. A strong noise level, at least in the autumn ice-free time period, practically leads to the impossibility of obtaining high-quality records of earthquakes. Ice-cover smooths out wind waves and significantly reduces noise level. Thus, it is recommended to conduct the OBS recording on the Arctic shelf in ice-covered time periods. For this, it is necessary to deploy the OBS in September–October and to dismantle in a year, or at least in June. At the same time, the requirements for the reliability of the power supply and dismantling mechanisms are significantly increased. The additional possibility of trawling stations in such conditions is quite useful. Our experience has shown high efficiency of this method of the OBS dismantling on the shelf after an annual setting. However, the risks of losing OBS are quite high, especially in shallow waters due to the presence of stamukhi.

The main characteristics of the broadband MET sensors, such as permissible installation angles, temperature range, sensitivity, dynamic range and frequency band, appeared to be suitable for obtaining the OBS records under the Arctic conditions to solve the seismological problems. The OBS deployments in the Laptev Sea resulted in a significant number of clear teleseismic arrivals, both body waves and surface waves within a broad frequency range. It allows conducting structural studies, for example with such approaches as the receiver function technique or surface wave group velocity inversion. For these methods, useful teleseismic waves have frequencies in the range of 0.1 to 1 Hz.

In addition to teleseismic signals, we have received a significant number of short-period signals from local microearthquakes. In tectonically active regions, such as the Laptev Sea region, the local microearthquakes’ distribution is crucial for describing present seismotectonic processes and for seismic hazard assessment. Another application of the OBS recordings for seismic hazard assessment is the site response analysis. Since this is a spectral approach, a wide frequency range is required. The obtained OBS records demonstrated peculiarities of the offshore site response analysis, such as the decrease in response at the frequencies close to the P-wave resonant frequency, caused by the water layer above the OBS.

Seismological applications highlighted in the paper have promising first results. Each topic can be further developed as a separate in-depth study. Especially, it concerns the possible link between the tectonic processes and bottom-up progressive thawing of subsea permafrost, which can accelerate massive methane release in the Siberian Arctic Seas [5,44]. Recent studies show the “mixing” origin of this methane in the innermost shelf [45] and deep termogenic origin of the escaping gas in the outer shelf of the Laptev Sea [46]. New information on the microearthquakes’ distribution and the structure of the lithosphere in this area may shed light on the mechanisms of this phenomenon. Another promising direction is a deeper study of the relationship of microseisms with meteorological parameters, which will allow a more detailed description of the mechanisms of their generation.

In addition to the fundamental research, a purely engineering direction is no less important. The study of the peculiarities of the influence of the sea soils and water column on the propagating seismic waves will be useful in the future construction of the extracting infrastructure in the perspective Arctic regions. The described OBS have demonstrated efficiency in solving a wide range of seismological problems and will be actively used in further Arctic expeditions.

## Figures and Tables

**Table 1:** Technical parameters of CME-4111 and CME-4311 seismometers.

Parameter	CME-4111	CME-4311
Type	No feedback	Vertical, with feedback, Horizontal, no feedback
Sensitivity	4000 V/(m/s)	2000 V/(m/s)
Type of output signal	analog, differential	analog, differential
Number of orthogonal components	3	3
Maximum output signal	±20 V	±10 V
Maximum input signal	±5 mm/s	±5 mm/s
Bandwidth	0.0083 (120 s) to 50 Hz	0.0167 (60 s) tp 50 Hz
Power supply voltage	12 V DC (10.5–16 V acceptable)	12 V DC (9.5–16 V acceptable)
Current consumption	7 mA	8.5 mA
Output impedance	2 × 500 Ohm	2 × 500 Ohm
Self-noise	see Figure 4	see Figure 4
Dynamic range at 1 Hz	123.5 dB	123.5 dB
Nonlinearity at 1 Hz	0.5%	Vertical, 0.15% Horizontal, 0.5%
Maximum inclination during installation	±15°	±15°
Temperature range	−12–+55 °C	−12–+55 °C
Housing material	Stainless steel (optional)	Stainless steel (optional)
Housing dimensions, diameter/height	146/90 mm	146/90 mm
Weight	3.1 kg	2.6 kg
Connector type on the housing to connect the cable	IDCC-10MR (10 pin)	IDCC-10MR (10 pin)

**Table 2:** Basic parameters of the Hydrophone 5007 m.

Frequency Band	0.04–2500 Hz
Sensitivity at 15 Hz	7.2 ± 0.5 mV/Pa
Dynamic range	100 dB
Sensor diameter	50 mm
Maximum depth	up to 5000 m

**Table 3:** Main characteristics of the recorder URS-S.

A number of Analog Channels	4 (Basic), 8 (Extended)
A number of digital channels	1 (for reference to absolute time)
Sample rates, Hz	20, 25, 40, 50, 80, 100, 160, 200, 400, 800
Time synchronization	GPS interface
Temperature stability of the quartz generator	±5 × 10^−9^
Dynamic range	85–90 dB
Memory	SD card up to 64 Gb
Power supply voltage	6.5–32 V
Power consumption	20 mA (12 V)

**Table 4:** Main technical parameters of the CME-3311 seismometer.

Frequency Band	1–50 Hz
Sensitivity	2000 V/(m/s)
Dynamic range at 1 Hz	118 dB
Power supply voltage	12V (10.5–16 V acceptable)
Current consumption	25 mA
Temperature range	−12–+55 °C
Maximum inclination during installation	±15°

**Table 5:** General characteristics of the GNS-C ocean-bottom station.

Deep-Water Radio Transparent Instrument Case	Glass Sphere of 43 cm Diameter Inlaid with Durable Plastic Shell
Seismic recorder	4 channels, 24-bit ADC for each channel
User interface/data backup link	Wireless USB. OS Windows client program for the device management
User terminal	Tablet, Laptop or Desktop PC
Seismic sensors	Molecular electronic sensors 3C, 0.0083 (120 s) to 50 Hz, one deep-water low-frequency hydrophone (0.067 Hz (15 s) to 30 kHz)
Release command	Acoustic call or automatically by preset time
Ballast anchor release	Electrochemical with mechanical support (salt and fresh waters)
Ballast anchor environmental feature	Self-dissolving into natural sea components after surveying (24 kg weight in air) or metallic
Continuous recording time	Up to 13 months (lithium batteries)
Battery type	3.6 V Primary lithium-thionyl chloride (Li-SOCl2). High-energy D-size cell-type LS 33,600 and battery protection board
Detecting equipment on the sea surface	Radio beacon with GPS coordinates transmission, flash light (at night), flag
Weight	38 kg in air (without anchor)
Depth range	up to 6000 m

**Table 6:** Main technical features of the hydrophone produced by the EDBOE RAS.

Sensitivity with Preamplifier	200 V/bar
Frequency range	From 0.067 Hz (15 s) to 30 kHz
Self-noise to input	Mean square noise in a range of 1 Hz to 1 kHz, 0.06 μBar
Maximal operation depth	6000 m
Physical size	4.2 cm long and 4.0 cm in diameter

**Table 7:** Main technical characteristics of the GNS recorder.

A Number of Input of Differential Channels	4
Supported data sampling	0.25, 0.5, 1, 2, 4 ms
Dynamic Range	153 dB
Time synchronization	Built-in GPS/GLONASS navigation receiver, powered off during recording
Temperature stability of the quartz generator	± 5 × 10^−9^
Memory	SD flash card of 32 Gb (expandable up to 128 Gb)
Power supply voltage	8.5–20 V
Main user interface	Wireless USB (non-active during data acquisition)

**Table 8:** Additional GNS-C sensors.

Temperature Measurement	2 Temperature Sensors
Current measurement	AD8218 sensor
Compass	LSM 303DLHC (3D accelerometer)
Pressure	BMP180 (Digital Pressure sensor)
Wireless USB dongle for communication	Alerion Wireless USB
Magnetic switch on|off of the OBS	Gerkon KЭM-2A
GPS|GLONASS receiver	Trimble Silvana or uBlox UC530M

**Table 9:** The coordinates, types and dates of operation for the OBS on the shelf and continental slope of the Laptev Sea in 2018–2020 (for the station locations, see Figure 9).

Site	Type	Latitude	Longitude	Depth	Operation Period
St4	MPSSR	75.422° N	127.391° E	42 m	6 Oct 2018 to 8 Feb 2019
St5	MPSSR	75.431° N	129.132° E	40 m	6 Oct 2018 to 8 Mar 2019
Slope	GNS-C	77.308° N	120.610° E	350 m	15 Oct 2018 to 31 May 2019
St3	MPSSR	76.392° N	125.660° E	51 m	9 Oct 2019 to 5 Jan 2020
Typ2	Typhoon	76.834° N	127.688° E	61 m	10 Oct 2019 to 9 Feb 2020

**Table 10:** Remote earthquakes registered at St4 and St5 sites and checked by the AK135 Travel Time Tables (campaign 2018–2019).

Time, UTC	M	Latitude	Longitude	Depth, km	Distance, °		Region
					St4	St5	
2018-11-18 20:25:46.590	6.8	−17.87	−178.93	540	99.0	98.6	Fiji
2018-11-25 16:37:32.830	6.3	34.36	45.74	18	54.8	55.3	Iran
2018-11-30 17:29:29.330	7.1	61.35	−149.96	46.7	30.2	29.7	Alaska
2018-12-01 13:27:21.080	6.4	−7.38	128.71	136	82.9	82.9	Indonesia
2018-12-05 04:18:08.420	7.5	−21.95	169.43	10	100.9	100.6	New Caledonia
2018-12-20 17:01:55.150	7.3	55.10	164.70	16.56	24.7	24.4	Russia
2019-01-05 18:47:11.740	5.9	51.33	−178.12	30	32.1	31.7	Alaska
2019-01-06 17:27:18.980	6.6	2.26	126.76	43.21	73.2	73.2	Indonesia
2019-01-08 12:39:30.950	6.3	30.59	131.04	35	44.9	44.9	Japan
2019-01-15 18:06:34.300	6.6	−13.34	166.88	35	92.0	91.7	Vanuatu
2019-01-22 05:10:03.480	6.3	−10.41	119.02	24	86.0	86.1	Prince Edward Islands region
2019-02-01 16:14:12.329	6.7	14.68	−92.45	66	86.7	86.4	Mexico
2019-02-02 09:27:36.030	6	−2.85	100.07	20	79.9	80.2	Indonesia

**Table 11:** The remote earthquakes registered at St3 and Typ2 sites and checked by the AK135 Travel Time Tables (campaign 2019–2020).

Time, UTC	M	Latitude	Longitude	Depth, km	Distance, °		Region
					St3	Typ2	
2019-11-20 08:26:08.017	6.3	53.13	153.68	496	25.6	25.6	Russia
2019-11-20 23:50:43.955	6.2	19.45	101.36	10	58.3	58.9	Thailand
2019-11-24 00:54:01.053	6.3	51.38	−175.51	20	33.3	33.0	Alaska
2019-11-23 12:11:15.564	6.2	1.64	132.81	5	74.9	75.2	Indonesia
2019-11-26 02:54:12.872	6.4	41.51	19.53	22	53.5	53.7	Mamurras
2019-12-02 05:01:54.821	6	51.19	−178.10	28	32.9	32.6	Amatignak
2019-12-15 06:11:51.155	6.8	6.70	125.17	18	69.7	70.1	Philippines
2019-12-20 11:39:52.874	6.1	36.54	70.46	212	46.6	47.3	Afghanistan
2019-12-23 20:56:23.555	6	50.52	−129.76	10	44.6	43.9	Canada
2019-12-23 19:49:43.086	6	50.61	−129.94	10	44.4	43.8	Canada
2019-12-23 19:13:25.075	5.7	50.54	−129.83	10	44.5	43.9	Canada
2019-12-25 03:36:01.626	6.3	50.61	−129.96	6.58	44.4	43.8	Canada
